# Supplementary material for: Structural order differentiation unlocks the energy storage performance of commensurate antiferroelectric ceramics
Source: Nat Commun. 2025 Oct 31;16:9660. doi: 10.1038/s41467-025-65321-x (PMC12578854; doi:10.1038/s41467-025-65321-x)
Supplement: Supplementary file 1 — Supporting Information [file 41467_2025_65321_MOESM1_ESM.pdf]

Supporting Information

Structural order differentiation unlocks the energy storage performance of commensurate antiferroelectric ceramics

Guanglong Ge [1, §], *Jin Qian* [1, §], *Cheng Shi* [1], *Chao Sun* [1], *Simin Wang* [1], *Hongguang Wang* [2, *], *Tengfei Hu* [3, 4, *], *Peter A. van Aken* [2], *Bo Shen* [1] *and Jiwei Zhai* [1, *]

[1]Key Laboratory of Advanced Civil Engineering Materials of Ministry of Education, Functional Materials Research Laboratory, School of Materials Science and Engineering, Tongji University, Shanghai 201804, China.

[2]Max Planck Institute for Solid State Research, 70569 Stuttgart, Germany.

[3]Shanghai Institute of Ceramics, Chinese Academy of Sciences, Shanghai 200050, China.

[4]School of Chemistry and Material Science, Hangzhou Institute for Advanced Study, University of Chinese Academy of Sciences, 1 Sub-lane Xiangshan, Hangzhou 310024, China.

[§]These authors contributed equally to this work.

*Corresponding author: hgwang@fkf.mpg.de; Hutengfei@mail.sic.ac.cn; apzhai@tongji.edu.cn

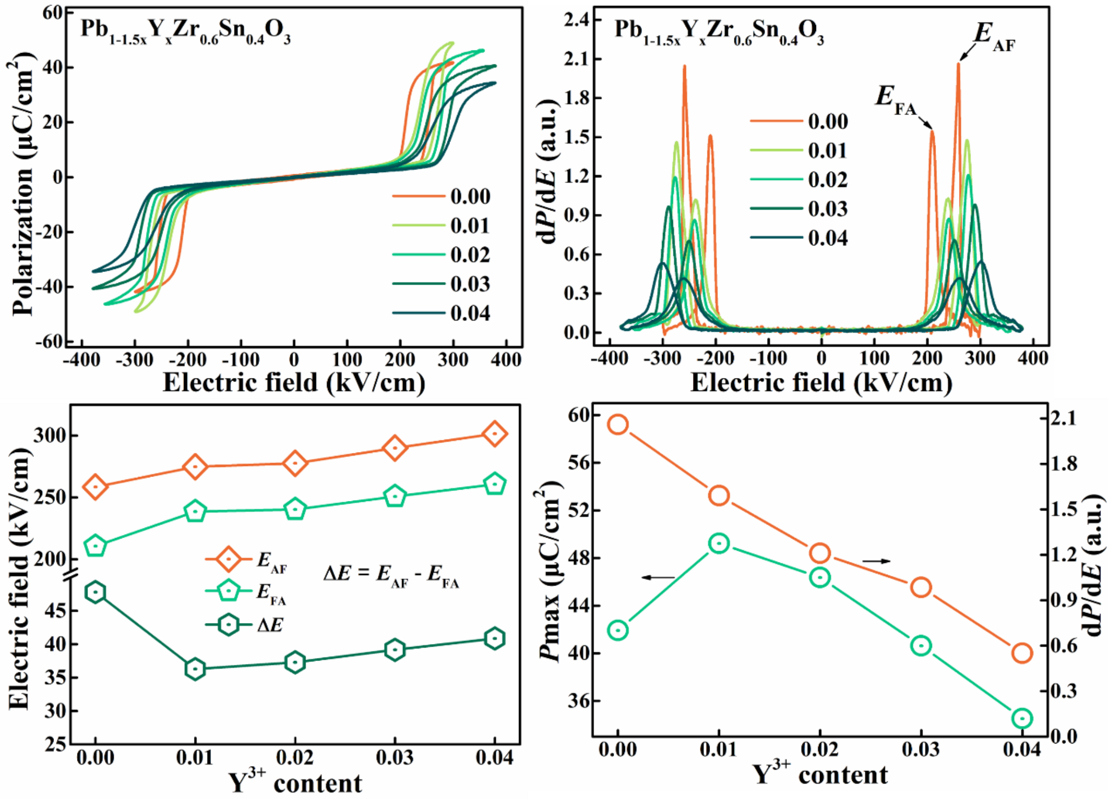

Figure S1. The room-temperature *P-E* loops, *J-E* curves, and the electrical parameters of $Pb_{1-1.5x}Y_xZr_{0.6}Sn_{0.4}O_3$ system with Y^{3+} content of 0~4 mol%.

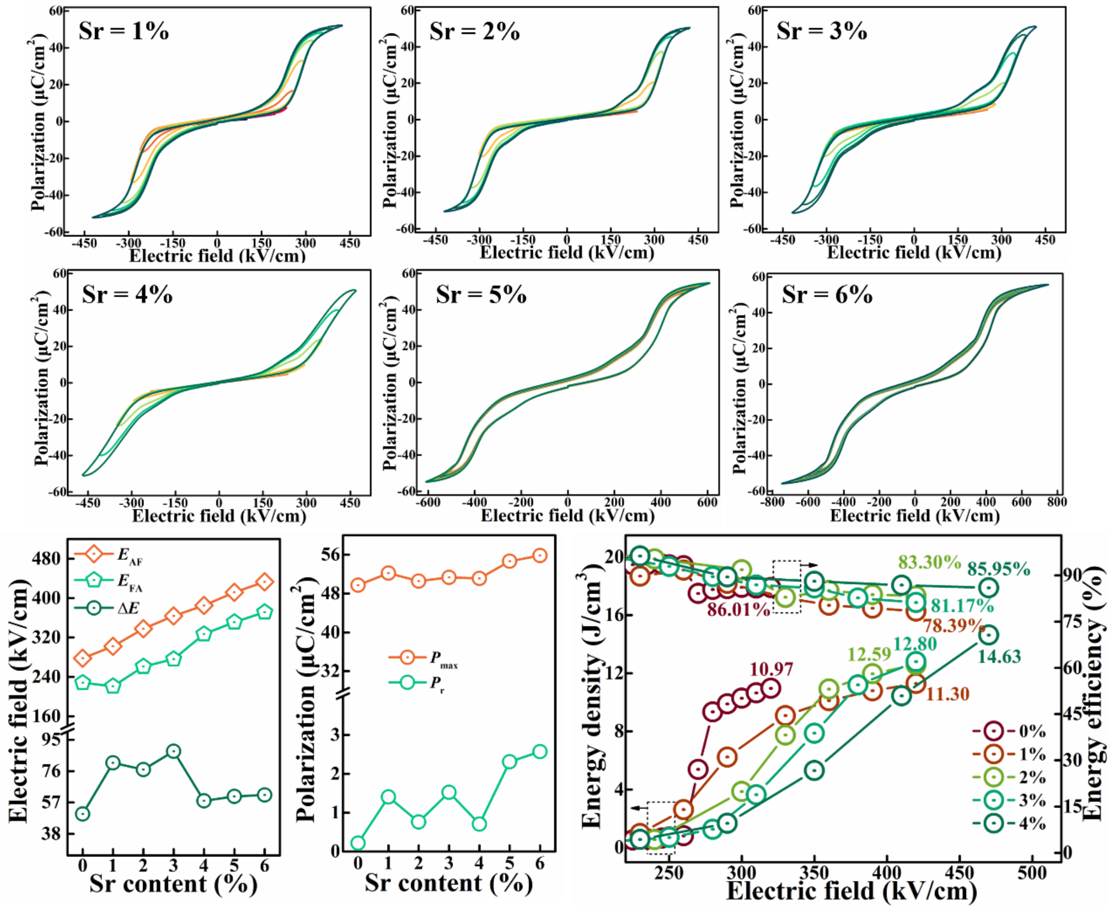

Figure S2. The room-temperature *P-E* loops, the electrical parameters and the energy storage properties of commensurate modulated AFE phases with Sr content less than 4 mol%.

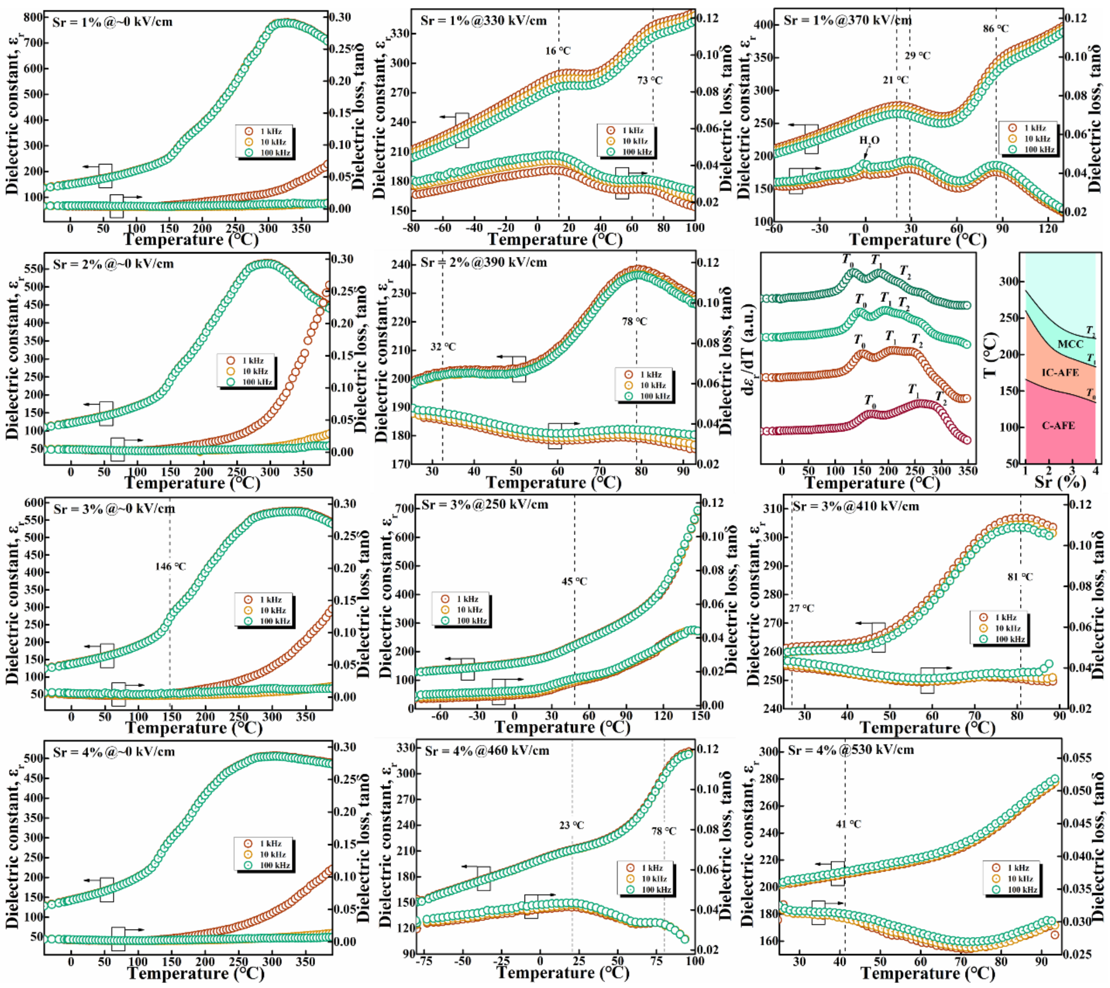

Figure S3. Temperature-dependent dielectric constant curves under different bias, the $(d\varepsilon_r/dT)-T$ curves, and the composition-temperature phase diagram.

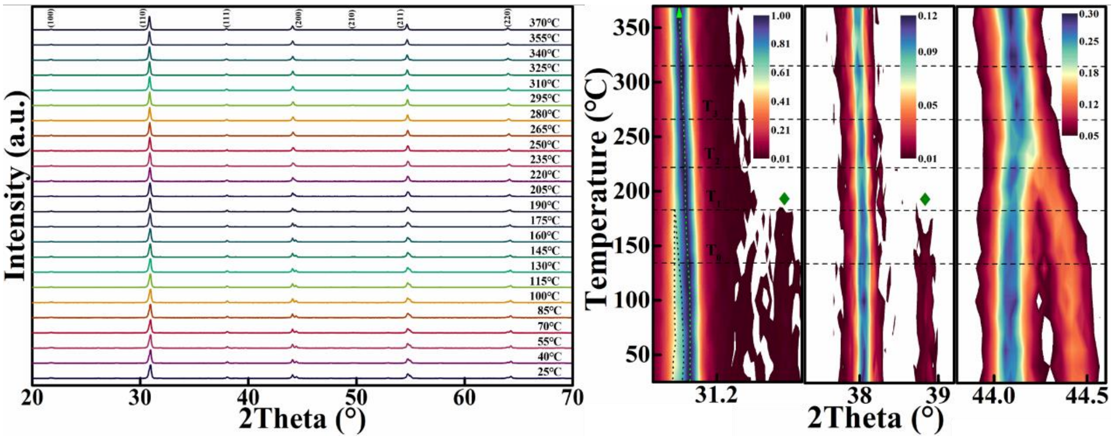

Figure S4. The temperature dependent XRD patterns of antiferroelectric S4.

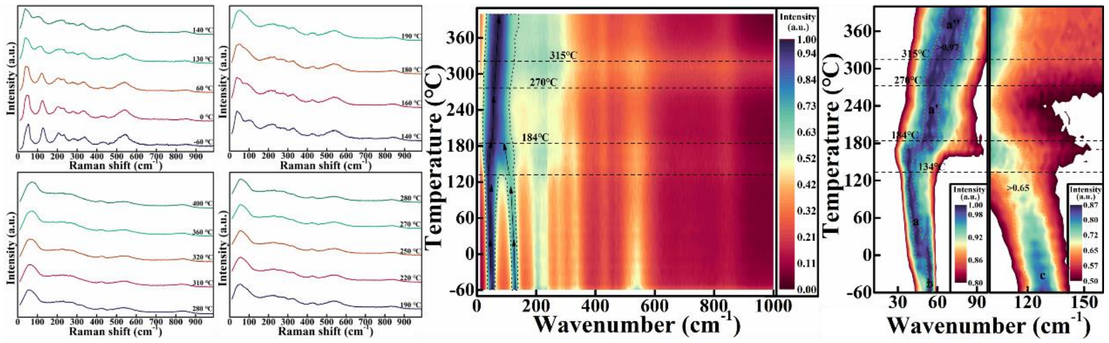

Figure S5. The temperature dependent Raman spectra of antiferroelectric S4 over the temperature range of -60~400 °C.

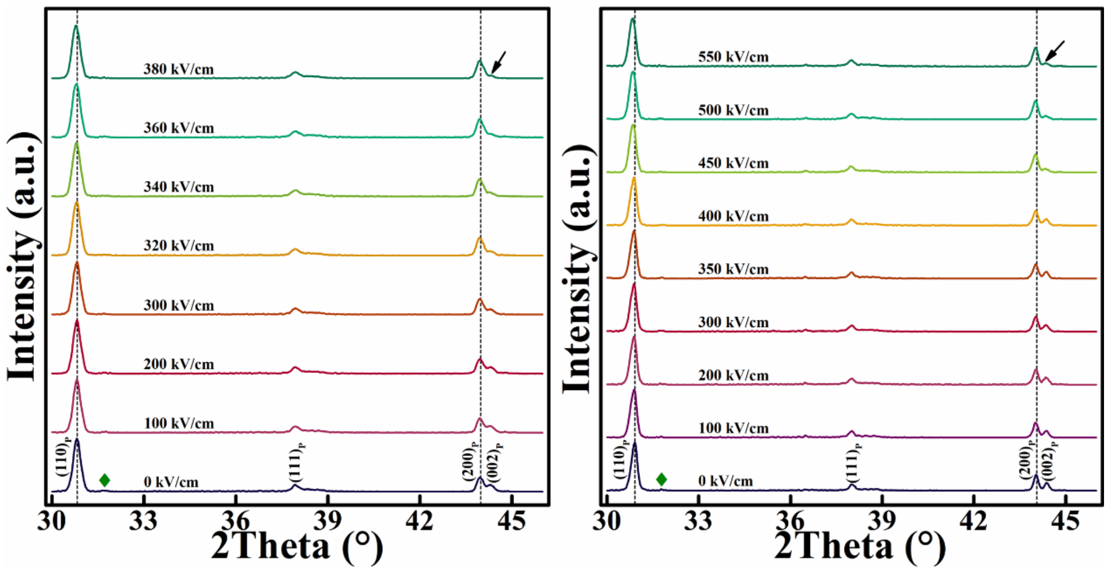

Figure S6. In-situ electric field-dependent XRD patterns of antiferroelectric S1 and S4 at room temperature.

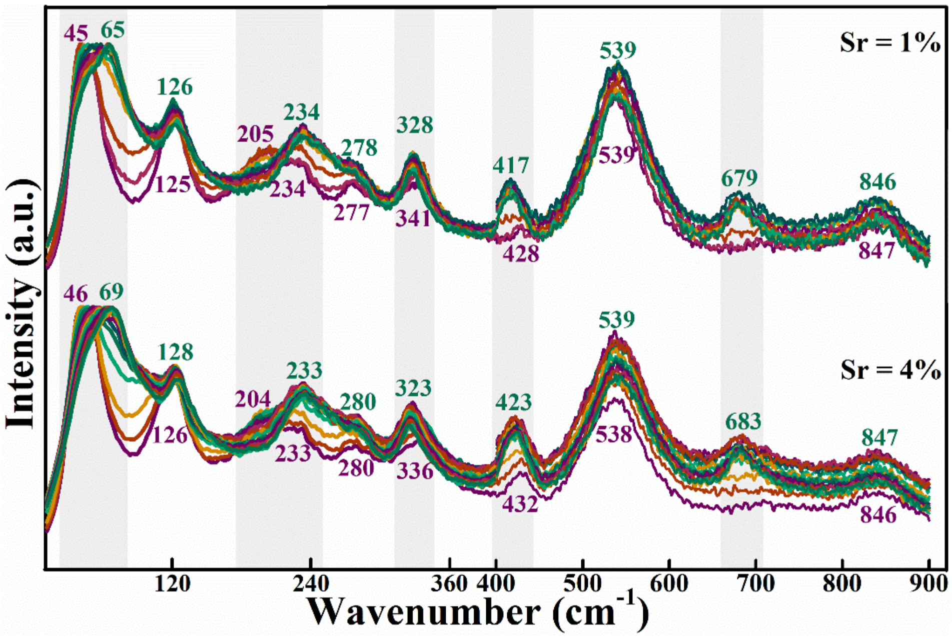

Figure S7. In-situ electric field-dependent Raman spectra of antiferroelectric S1 and S4 at room temperature.

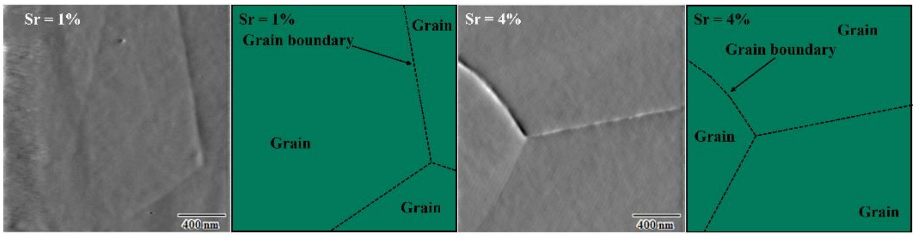

Figure S8. The PFM surface morphology (2 μm *2 μm) of antiferroelectric S1 and S4.

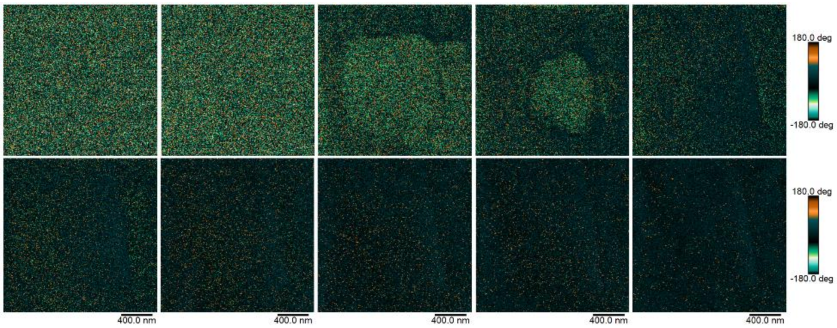

Figure S9. The PFM phase distribution of S1 at different bias electric field.

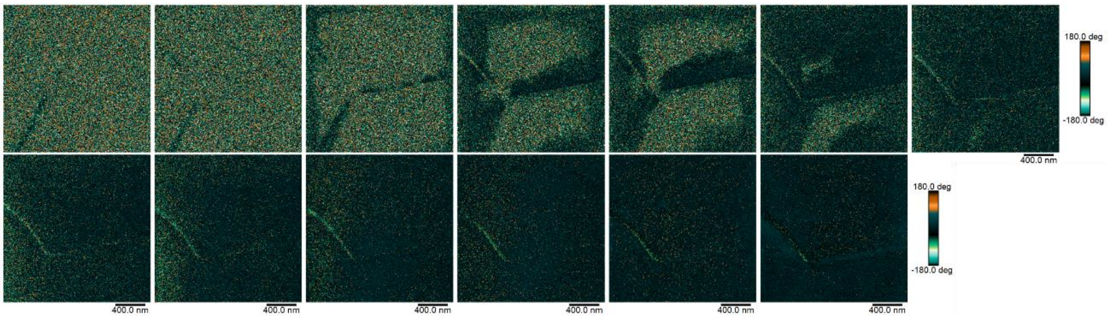

Figure S10. The PFM phase distribution of S4 at different bias electric field.

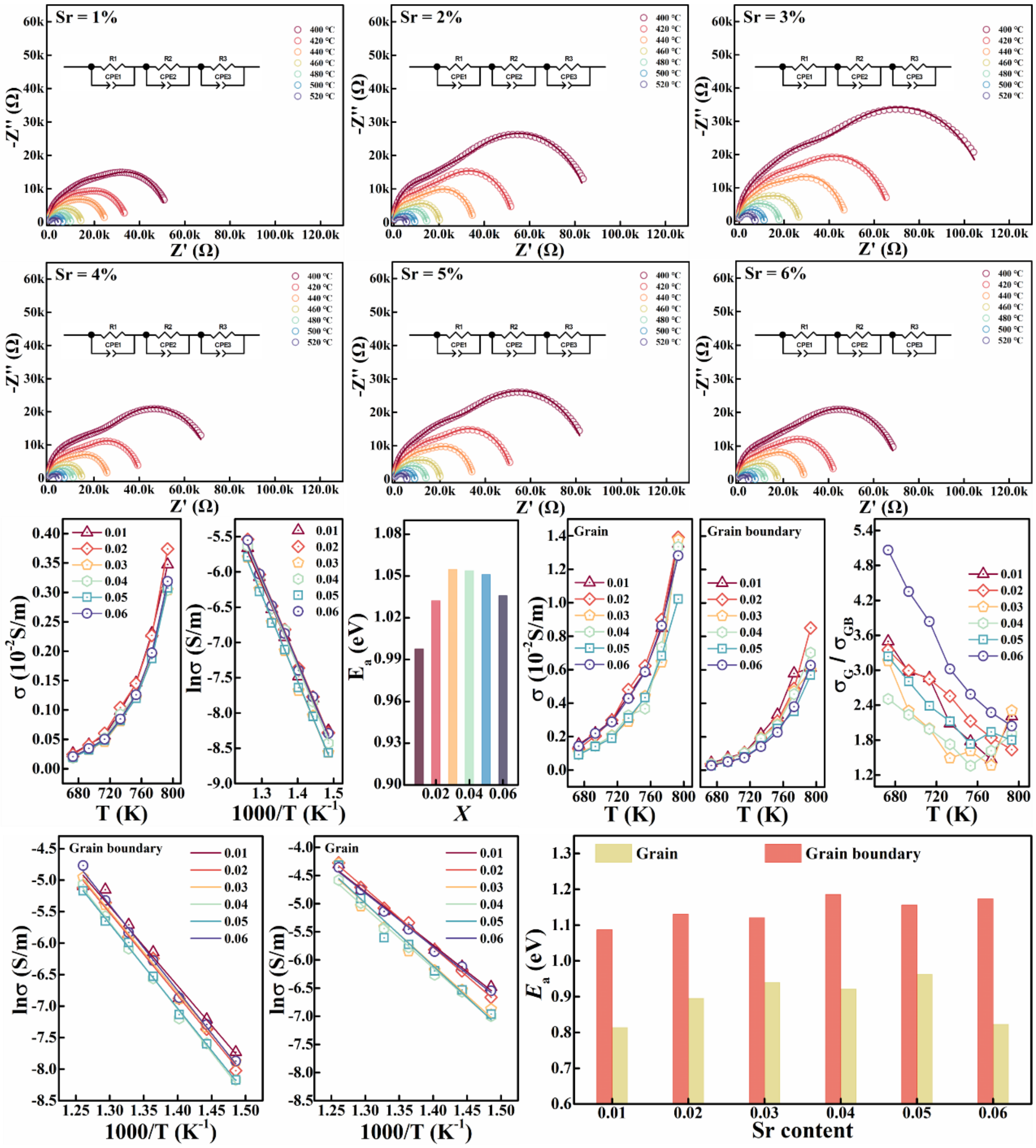

Figure S11. The high temperature complex impadence spectroscopy of PSY system.

It is evident in the Nyquist or Cole-Cole plots that the approximate semicircle arc consisting of several frequency-dependent complex impedance responses can be observed. At the high frequency regions, there was a weak impedance response process, where the intersection of the high frequency end and the real axis was close to the zero point, indicating that the high frequency part of the impedance spectra came from the bulk response. As the temperature increases from 400 to 520 °C, the impedance semicircles shrink successively, which is a characteristic of a thermally activated relaxation process. Using ZView 2.0, all results are fitted using (RQ)(RQ)(RQ) equivalent circuit, each consisting of a parallel ideal resistor R and a constant phase

element Q or CPE. Three series of (RQ) units respectively belong to the electrical response of grain boundary, grain, and the polarization of electrodes. The fitting results show small chi-squared function (χ^2) on the order of 10^{-4}, as well as small error of less than 3% for each unit component. The resistance of grain boundaries, grains, and electrodes are labeled here as R_{GB}, R_G, and R_e respectively, which are usually located in the mid-frequency, high-frequency, and low-frequency regions, and their values decrease in sequence. For bulk conductivity behavior, it is evident that the conductivity can be divided into two levels with Sr doping. The conductivity of Sr1 and Sr2 is higher throughout the entire temperature range, indicating less ideal electrical insulation. Sr3-Sr6 show lower conductivity and thereby their breakdown electric field should be higher than that of Sr1-Sr2. We also separate the contributions of grain boundaries and grains to conductivity, as shown in the middle right figure. Overall, the conductivity of grains is two or three times higher than that of grain boundaries. And as the temperature increases, the values of the two become comparable. Thus, it can be inferred that the impedance at room temperature is mainly provided by grain boundaries, which is in line with expectations. For Sr3-Sr5, the contribution of grains on impedance is higher than that of Sr1-Sr2 and Sr6, while the contribution of grain boundaries of Sr5-Sr6 is higher than the rest compositions. This implies that for polymorphic modulated AFE phase, the coexisting of commensurate and incommensurate modulated AFE phases does benefit the bulk insulation. Using the Arrhenius equation , we can calculate the activation energy (E_a) related to the temperature dependent impedance response. For a pure commensurate modulated AFE matrix, i.e. Sr0-Sr4, the E_a increases with Sr doping content from 0.99 to 1.05 eV. As we all know, in a ABO_3 perovskite structure the A- and B-site cations respectively display E_a of ~4 and ~12 eV, while that of oxygen vacancies is varied from 0.5 to 2 eV depending on their concentration. Thus, the formation and migration of oxygen vacancies contribute mainly to the ceramic conductivity. We also know that a A-site equivalent substitution mode will not fundamentally produce more oxygen vacancies, and thus the increased E_a should be related to the migration of oxygen vacancies. Therefore, the increase in the E_a from 0.99 to 1.05 eV for the pure commensurate modulated AFE ceramics indicates that carrier

migration necessitates overcoming a higher energy barrier. And in PSY1 system more Sr doping content leads to a decreased cell volume, which limits the migration of oxygen vacancies and should be the main reason for enhanced E_a. However, for the polymorphic modulated AFE Sr5-Sr6, their E_a decreases with further doping. For Sr5-Sr6, the potential inhomogeneity caused by polymorphic modulated AFE coexistence forms grain boundary and plays a major role in the contribution of conductivity. Therefore, it is not difficult to imagine that for PSY1 system, although a composition induced phase transition occurs with increasing Sr doping, the changes in bulk conductivity are still understandable. The impedance of commensurate modulated AFE phase increases with Sr doping, mainly due to the enhanced E_a at both grain boundaries and grains. When transformed into polymorphic modulated AFE, the competition between grain and grain boundary causes a higher contribution of grain boundaries to bulk conductivity, which enables the overall insulation to be improved by ignoring the reduced E_a of grains. That is, the factors affecting the breakdown characteristics of the PSY1 system can be summarized as being determined by the E_a, the grain/grain boundary conductivity, and the balance between grain and grain boundaries as a whole. Eventually, phase region end composition Sr4 possesses both lowest grain/grain boundary conductivity as well as highest grain/grain boundary activation energy, it should perform a better breakdown performance than Sr0-Sr3 and is selected as the optimal composition when jointly considering its highly differentiated structural order. Of course, the breakdown electric field of Sr5 and Sr6 can be higher, but this is no longer within the view of pure commensurate modulated AFE phase and will not be further verified here.

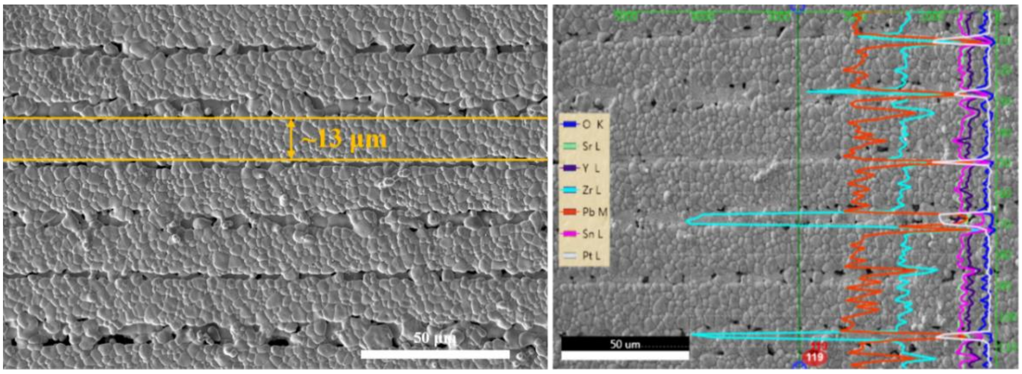

Figure S12. The cross-sectional SEM images and the element distribution of S4 MLCCs. The thickness of monolayer is about 13 μm, and there is a certain overlap between the platinum slurry and the ceramic in the Zr element distribution as the Pt-75 slurry contains a certain amount of ZrO_2 particles to meet the requirements of high-temperature sintering.

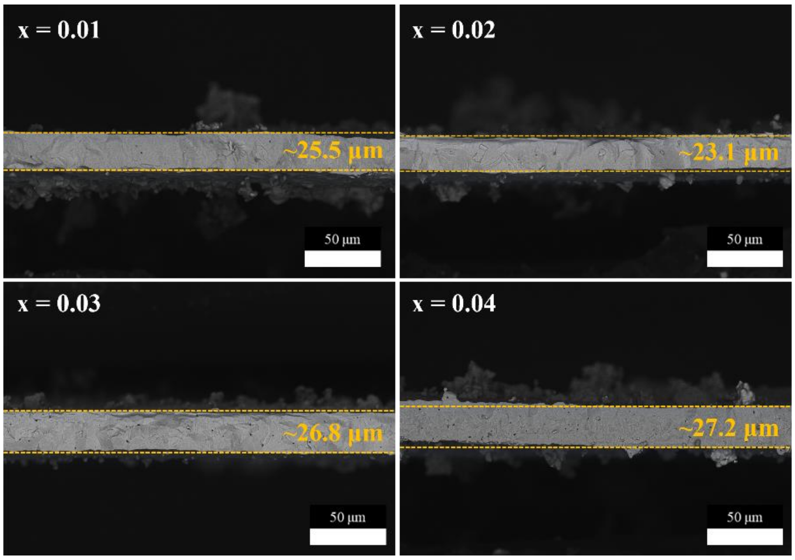

Figure S13. The cross-sectional SEM images of PSY1 system.
